# Supplementary material for: Transcriptomic differences between human 8-cell-like cells reprogrammed with different methods
Source: Stem Cell Reports. 2023 Jul 20;18(8):1621–8. doi: 10.1016/j.stemcr.2023.06.009 (PMC10444576; doi:10.1016/j.stemcr.2023.06.009)
Supplement: Document S1. Figures S1–S4 [file mmc1.pdf]

Stem Cell Reports, Volume *18*

Supplemental Information

Transcriptomic differences between human 8-cell-like cells reprogrammed with different methods

Masahito Yoshihara and Juha Kere

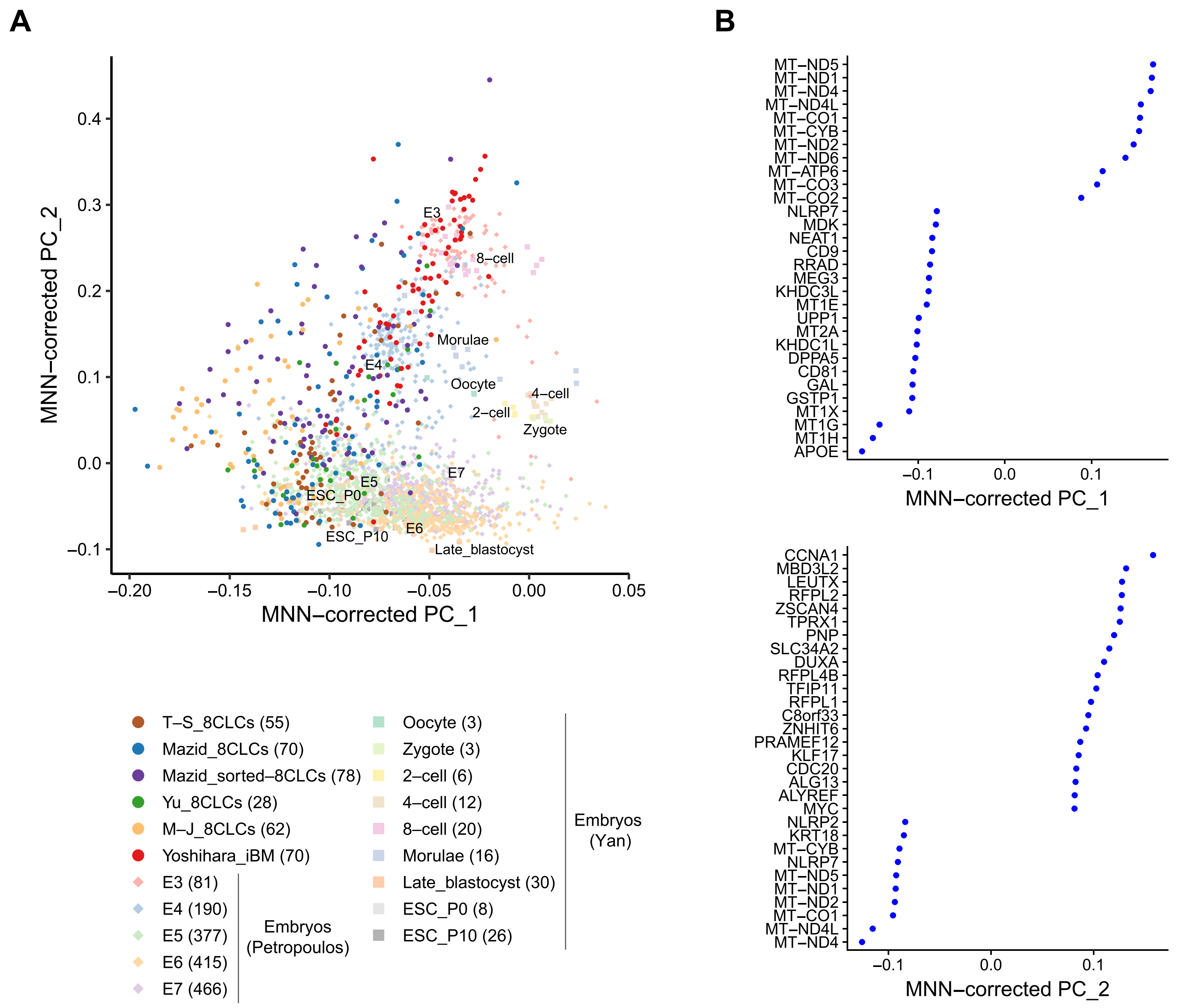

Figure S1. MNN-corrected PCA of human 8CLCs with pre-implantation embryos, related to Figure 1

(A) A PCA plot colored by the original cell types. T-S and M-J stand for Taubenschmid-Stowers and Moya-Jódar, respectively. E3–7, embryonic day 3–7; P0, passage 0; P10, passage 10. Numbers in parentheses represent the numbers of analyzed cells.

(B) Top 30 genes contributing to the MNN-corrected principal components (PCs) 1 and 2.

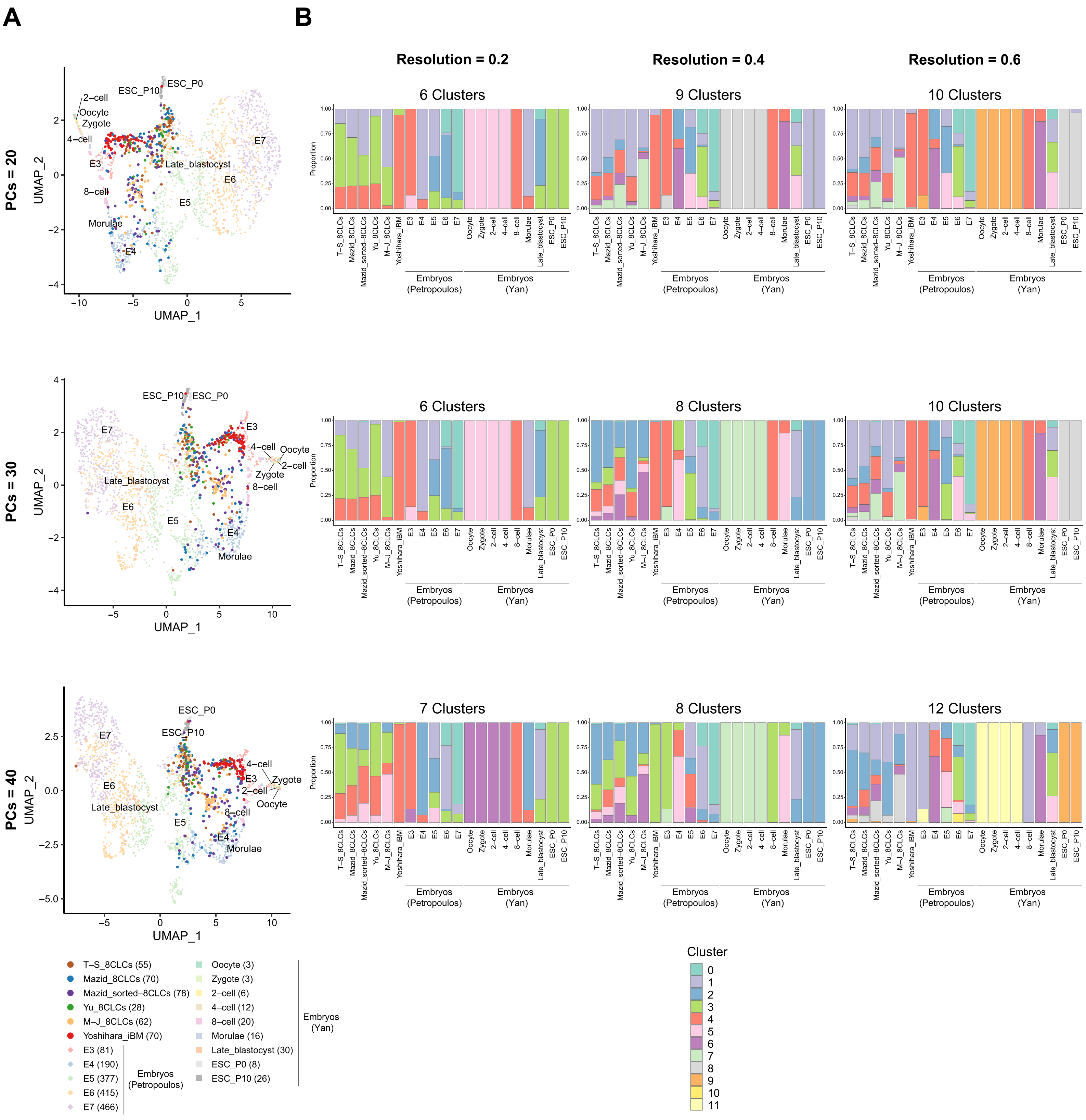

Figure S2. Unsupervised clustering of human 8CLCs and pre-implantation embryos with different parameters, related to Figure 1

(A) UMAP plots colored by the original cell types. UMAP was generated using the top 20, 30, or 40 MNN-corrected PCs.

(B) Bar plots show the proportion of each cluster in each cell type. Clustering was performed with different resolutions (0.2, 0.4, or 0.6) for each parameter shown in A.

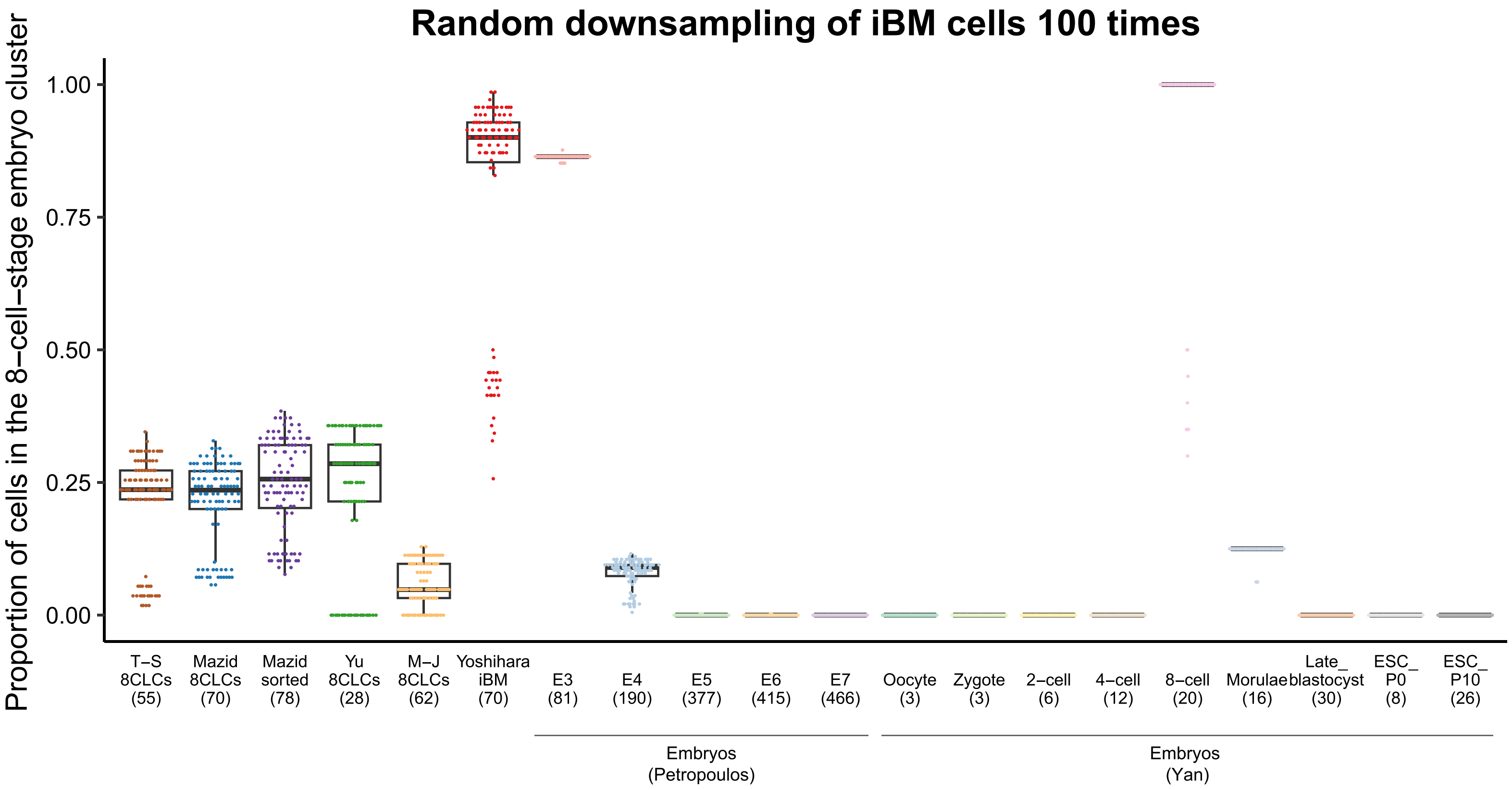

Figure S3. The proportion of cells clustered with 8-cell-stage embryos after random downsampling of iBM cells 100 times, related to Figure 1

iBM cells of Yoshihara were randomly picked 100 times and the proportion of cells clustered with the E3 or 8-cell-stage cells was calculated. Each dot represents one iteration.

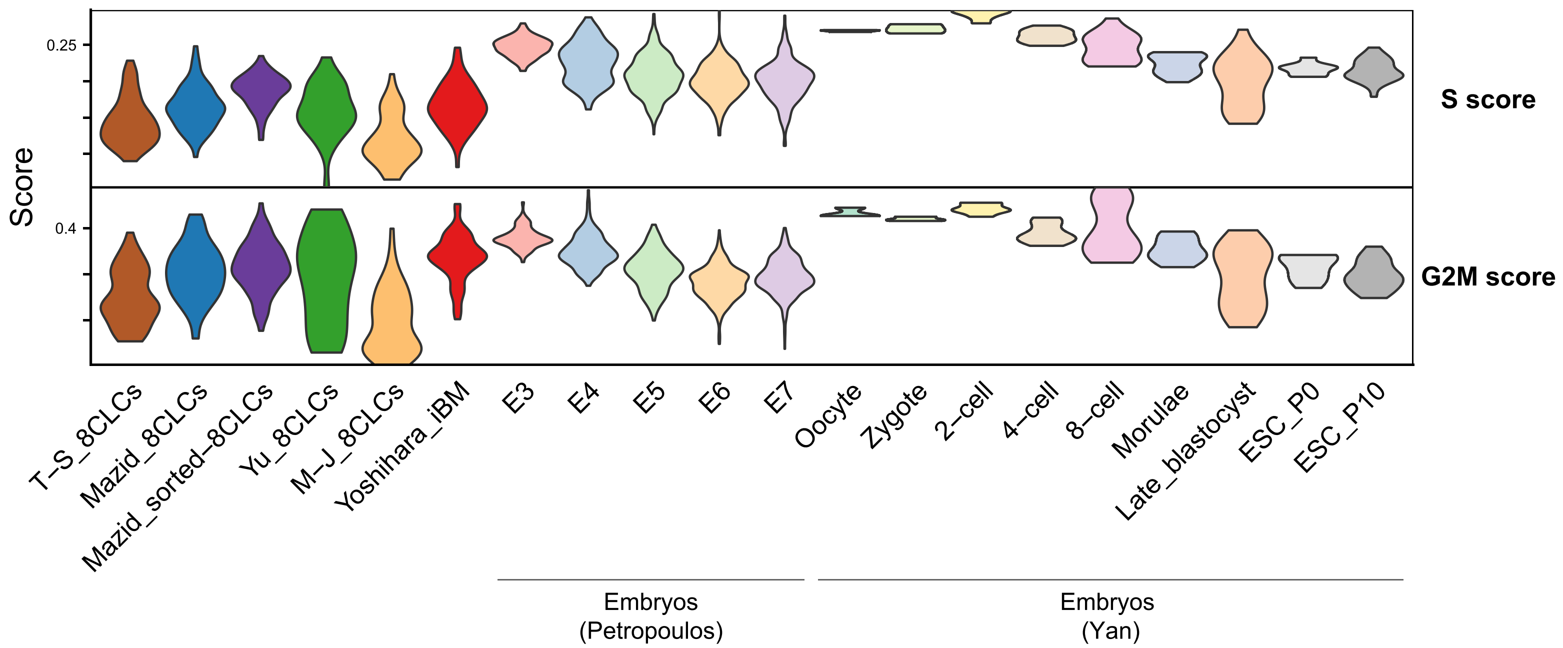

Figure S4. Violin plots show the cell cycle scores, related to Figure 4
