# Supplementary material for: Transcriptomic differences between human 8-cell-like cells reprogrammed with different methods
Source: Stem Cell Reports. 2023 Jul 20;18(8):1621–8. doi: 10.1016/j.stemcr.2023.06.009 (PMC10444576; doi:10.1016/j.stemcr.2023.06.009)
Supplement: Document S2. Article plus supplemental information [file mmc2.pdf]

Stem Cell Reports
Report
OPEN ACCESS

Transcriptomic differences between human 8-cell-like cells reprogrammed with different methods

Masahito Yoshihara[1,2,3,*] and Juha Kere[1,4,5,*]
[1]Department of Biosciences and Nutrition, Karolinska Institutet, Stockholm, Sweden
[2]Institute for Advanced Academic Research, Chiba University, Chiba, Japan
[3]Department of Artificial Intelligence Medicine, Graduate School of Medicine, Chiba University, Chiba, Japan
[4]Folkhälsan Research Center, Helsinki, Finland
[5]Stem Cells and Metabolism Research Program, University of Helsinki, Helsinki, Finland
*Correspondence: masahito.yoshihara@chiba-u.jp (M.Y.), juha.kere@ki.se (J.K.)
https://doi.org/10.1016/j.stemcr.2023.06.009

SUMMARY

Embryonic genome activation (EGA) is a critical step in embryonic development. However, while EGA has been studied in mice using mouse 2-cell-like cells, human EGA remains incompletely elucidated due to the lack of an *in vitro* cell model recapitulating the early blastomere stage in humans. Recently, five groups independently reported human 8-cell-like cells (8CLCs, also called induced blastomere-like cells) developed from pluripotent stem cells and used single-cell RNA sequencing (scRNA-seq) to specify their cellular identities. Here we summarize the methods developed to produce the 8CLCs and compare their transcriptomic profiles by integrating them with the scRNA-seq datasets of human embryos. These observations will allow comparison and validation of the models, stimulate further in-depth research to characterize the genes involved in human EGA and pre-implantation development, and facilitate studies on human embryogenesis.

INTRODUCTION

Embryonic genome activation (EGA) is an essential process in the initiation of an organism's development. However, the regulatory mechanisms of EGA in humans are still largely unknown due to the very limited availability of cells for research and also ethical concerns regarding the beginning of life. Therefore, cell models mimicking human blastomeres are highly needed to study the earliest stages of human EGA, to clarify the roles of early transcription factors, and to understand the events that occur in the early embryo. Mouse embryonic stem cells (ESCs) spontaneously but rarely form a population of 2-cell-like cells (2CLCs) that exhibit characteristics similar to 2-cell-stage mouse embryos (Macfarlan et al., 2012). Although mouse 2CLCs have an expanded cell fate potential that can contribute to both embryonic and extra-embryonic lineages, they have limited stability and are prone to transition back to the pluripotent state (Genet and Torres-Padilla, 2020).

Recently, five research groups independently developed methods to produce 8-cell-like cells (8CLCs, also called induced blastomere-like [iBM] cells) from human pluripotent stem cells (hPSCs) (Mazid et al., 2022; Moya-Jódar et al., 2023; Taubenschmid-Stowers et al., 2022; Yoshihara et al., 2022; Yu et al., 2022). Although their specific approaches differed, all employed single-cell RNA sequencing (scRNA-seq) to identify the 8CLCs that emerged as small populations.

Taubenschmid-Stowers et al. (2022) identified a subpopulation of cells (~1.6%) exhibiting EGA gene upregulation in naive human ESCs (hESCs) cultured in PXGL medium: PD0325901 (a MEK inhibitor), XAV939 (a tankyrase inhibitor), Gö6983 (a PKC inhibitor), and human leukemia inhibitory factor [LIF]) (Guo et al., 2017). These cells were called 8CLCs. Like mouse 2CLCs, these 8CLCs spontaneously emerge from among naive hESCs. Single-cell assay for transposase-accessible chromatin with high-throughput sequencing (scATAC-seq) analysis revealed that these 8CLCs exhibited increased chromatin accessibility proximal to *ZSCAN4*, which was also seen in 4- and 8-cell-stage embryos. TPRX1 was used as the 8CLC marker, and *DUX4* overexpression in naive hESCs increased the number of TPRX1-positive cells.

Mazid et al. (2022) first optimized the culture conditions to generate naive hPSCs from primed hPSCs, using 4CL (four chemicals + human LIF): PD0325901, IWR1 (a tankyrase inhibitor), 3-deazaneplanocin A (DZNep; suppresses H3K27 methyltransferase EZH2), and trichostatin A (TSA; a class I histone deacetylase inhibitor), plus LIF. Next, they discovered that increased doses of DZNep and TSA (enhanced 4CL: e4CL) promoted the expression of EGA genes. Although both primed PSCs cultured for 12 days in 4CL followed by 5 days in e4CL (stepwise e4CL-D5) and those cultured directly in e4CL for 7 days expressed EGA genes, the stepwise e4CL-D5 cells showed higher similarity to 8-cell-stage embryos. scRNA-seq of the stepwise e4CL-D5 cells revealed that a subpopulation (11.9%) of cells resembled 8-cell-stage embryos called 8CLCs. scATAC-seq of the stepwise e4CL-D5 cells also identified 8CLCs with similar chromatin accessibility patterns as 8-cell-stage embryos. Furthermore, 8CLCs exhibited chromatin opening at 8-cell-stage-specific transposable

This is an open access article under the CC BY license (http://creativecommons.org/licenses/by/4.0/).

elements (TEs) such as MLT2A1 and LTR12C. These 8CLCs could be sorted using the TPRX1-EGFP reporter. Gene regulatory network analysis demonstrated that DPPA3 and TPRX1 play a central role in 8CLCs, and *DPPA3* or *TPRX1* knockout markedly downregulated the EGA genes, indicating that these 8CLCs are useful as a cell model to study EGA genes. Furthermore, these 8CLCs differentiated into trophoblast stem cells (TSCs) in the form of blastocyst-like structures (blastoids) (Yanagida et al., 2021) and teratomas.

Yu et al. (2022) first conducted chemical screening of hESCs and generated pre-implantation epiblast-like hPSCs (prEpiSCs), which showed a greater similarity to pre-implantation epiblast cells than other naive hPSCs such as those cultured with five inhibitors, LIF, and activin (5iLA) (Theunissen et al., 2014). By performing scRNA-seq of prEpiSCs, they identified a rare cell population that expressed EGA genes and named these cells 8CLCs. They isolated the 8CLCs from prEpiSCs with the 8-cell-specific LEUTX-mCherry reporter. They further identified small molecules that promote the conversion of prEpiSCs to 8CLCs: DZNep, GSK1120212 (a MEK inhibitor), XAV939, AG14361 (a PARP1 inhibitor), and CBL0137 (a p53 activator). In addition, two chemical compounds (GSK872 and Ac-DEVD-CHO) were applied to inhibit cell death. These converted 8CLCs were named chemical-induced 8CLCs (ci8CLCs) and could be maintained for 2–3 weeks. Importantly, ci8CLCs formed blastoids (Kagawa et al., 2022), and TSCs could be derived from these blastoids.

Yoshihara et al. (2022) transiently overexpressed *DUX4* in primed hESCs to avoid DUX4-mediated cytotoxicity (Bosnakovski et al., 2008). They found that EGA genes and 8-cell-stage-specific TEs such as MLT2A1, MLT2A2, and HERVL (Hendrickson et al., 2017) were significantly upregulated within 24 h after induction. scRNA-seq analysis of the hESCs treated with a 15-min pulse of *DUX4* demonstrated that 6.6% of the cells had a similar expression profile as 8-cell-stage embryos, at 12 h after induction. They named these cells iBM cells. Using fluorescence-activated cell sorting without a transgenic reporter construct, they enriched the viable iBM cells using an antibody against the cell surface antigen NaPi2b (SLC34A2), which is highly expressed in 8-cell-stage embryos but rarely expressed in hESCs. In one experiment, up to 17% of the *DUX4*-pulsed cell population expressed NaPi2b. In addition, the authors identified a subpopulation of cells, called "late 1" cluster cells, that appeared to be derived from iBM cells (obtained 24–48 h after induction). These "late 1" cluster cells showed high similarity to naive hESCs and early blastocysts.

Moya-Jódar et al. (2023) performed scRNA-seq on naive human induced PSCs (hiPSCs) cultured with 5iLAF (5iLA plus FGF2). They identified a subpopulation of cells (~1.7%) that expressed EGA genes and termed them 8CLCs. These 8CLCs also highly expressed 8-cell-stage-specific TEs such as MLT2A1. The authors integrated their scRNA-seq data with those of Taubenschmid-Stowers et al. and Mazid et al. and demonstrated their similarity.

All these 8CLCs differ from 8-cell-stage blastomeres due to their cellular background; instead of containing mRNA and proteins of oocyte origin (such as zona pellucida proteins), they carry over mRNA and proteins from the blastocyst-derived ESCs. Also, the mode of reprogramming, especially by different chemical factors or the EGA-initiating DUX4 protein, may lead to cells with somewhat different properties, even though they might all be identified as 8CLCs. An obvious question is how similar or different these 8CLCs are. A straightforward means of determining the answer involves assessing their expression profiles and clustering patterns and then comparing them both with each other and with embryonic 8-cell-stage blastomeres for which scRNA-seq data are available.

RESULTS

Integrative analysis of 8CLCs and pre-implantation embryos

To assess the differences between the 8CLCs and their similarities with human pre-implantation embryos, we obtained the scRNA-seq data of the five aforementioned studies and integrated them with two datasets of human pre-implantation embryos (Petropoulos et al., 2016; Yan et al., 2013). The Yan et al. dataset includes primed hESCs as well as embryos, whereas the Petropoulos et al. dataset includes samples starting from embryonic day 3 (E3, 8-cell stage) to 7 (E7). Here we refer to the different datasets by the names of the first author of each study (T-S and M-J stand for Taubenschmid-Stowers and Moya-Jódar, respectively).

Datasets of different batches were corrected using the mutual nearest neighbors (MNN) approach. MNN-corrected principal component analysis (PCA) indicated that mitochondrial gene expression distinguished 8CLCs and pre-implantation embryos (Figure S1). Notably, EGA genes highly contributed to the MNN-corrected PC_2, distinguishing 8-cell-stage embryos from those at other stages. Uniform manifold approximation and projection (UMAP) dimensionality reduction demonstrated that human embryos at similar stages across the two datasets (i.e., E3 and 8-cell, E4 and morulae, E6 and late blastocyst) were closely distributed, indicating successful integration (Figure 1A). Although the Mazid datasets were prepared with two different platforms, specifically droplet-based (stepwise

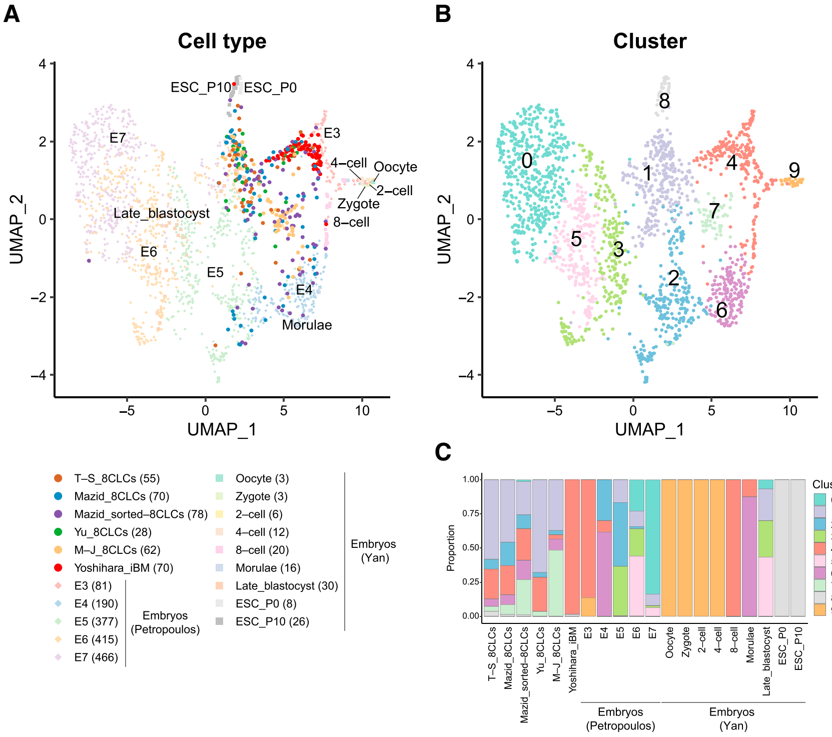

Figure 1. Unsupervised clustering of human 8CLCs with pre-implantation embryos
(A) A UMAP plot colored by the original cell types. T-S and M-J stand for Taubenschmid-Stowers and Moya-Jódar, respectively. E3–7, embryonic day 3–7; P0, passage 0; P10, passage 10. Numbers in parentheses represent the numbers of analyzed cells.
(B) A UMAP plot colored by Louvain clusters.
(C) Bar plots show the proportion of each cluster in each cell type. See also Figures S1–S3.

e4CL-D5) and SMART-seq2 (sorted 8CLCs), the cells showed similar distribution patterns. The iBM cells of Yoshihara were densely clustered, whereas the other 8CLCs tended to be broadly dispersed.

Unsupervised clustering resolved 10 clusters corresponding to embryonic developmental stages (Figure 1B). Oocytes, zygotes, and 2- and 4-cell-stage cells formed one cluster (cluster 9), as did primed hESCs (passages 0 and 10) (cluster 8) (Figure 1C). Cluster 6 consisted mainly of E4 and morulae, cluster 3 of E5, cluster 5 of E6 and late blastocysts, and cluster 0 of E7. Many of the 8CLCs other than Yoshihara iBM cells were classified into cluster 1 or 7. Cluster 1 also included blastocysts (E5–7 and late blastocyst), whereas cluster 7 comprised mostly 8CLCs. Notably, E3 and 8-cell-stage cells were classified into cluster 4, together with different proportions of the 8CLCs: 99% of the iBM cells of Yoshihara; 21%–25% of the 8CLCs of T-S, Mazid, and Yu; and 3% of the M-J 8CLCs. To confirm these findings, we repeated the dimensionality reduction and clustering processes with different parameters, and we consistently obtained similar results (Figure S2).

Because there were 1,528 iBM cells in the original Yoshihara dataset, and the other datasets reported 28–78 8CLCs, we randomly selected 70 iBM cells for the comparisons. To exclude sampling bias, we repeated the random sampling 100 times and found that ~90% of the iBM cells of Yoshihara, ~23%–29% of the 8CLCs of T-S, Mazid, and Yu, and ~5% of the 8CLCs of M-J clustered together with the 8-cell-stage cells (Figure S3). Therefore, of the 8CLCs, iBM cells were most similar to the 8-cell-stage embryo, whereas the other reported 8CLC populations were heterogeneous.

Cell type annotation of 8CLCs

We further annotated the 8CLCs using the scRNA-seq data of human pre-implantation embryos as references. Using the Petropoulos dataset as a reference, ~50% of the iBM cells of Yoshihara were annotated as E3, compared with 4%–11% of the 8CLCs of T-S, Mazid, and Yu, and none of the 8CLCs of M-J (Figure 2A). Using the Yan dataset as a reference, ~66% of the iBM cells of Yoshihara were annotated as 8-cell, compared with 7%–15% of the 8CLCs of T-S, Mazid, and Yu, and 2% of the 8CLCs of M-J (Figure 2B). Interestingly, a majority of the 8CLCs of T-S, Mazid, Yu, and M-J were annotated as E5 or late blastocyst, whereas most iBM cells not annotated as E3, or 8-cell were annotated as E6 or ESC (passage 10). When the naive and primed PSCs (Messmer et al., 2019) were annotated using the Petropoulos dataset as a reference, most naive PSCs and primed PSCs were annotated as E5 and E6, respectively (Figure 2A). These observations may reflect the fact that iBM cells were derived from primed hESCs, whereas the other 8CLCs were derived from naive hPSCs.

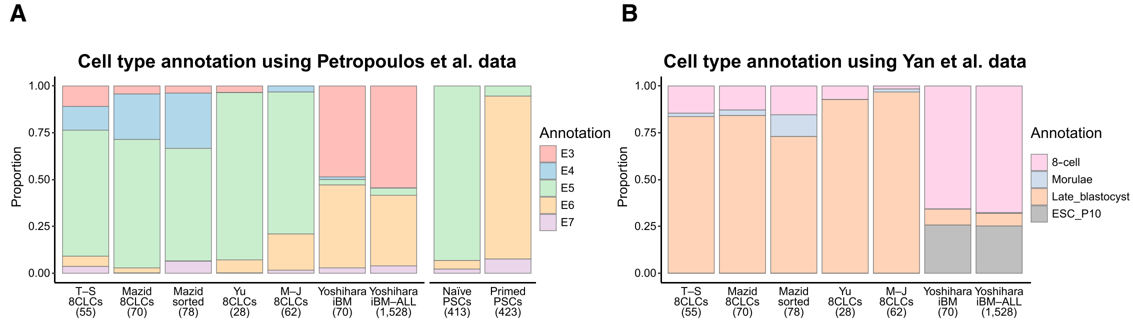

Figure 2. Cell type annotation of human 8CLCs with pre-implantation embryos

(A) Cell type annotation of human 8CLCs (left) and human naive and primed PSCs (right) using Petropoulos et al. data as a reference.

(B) Cell type annotation of human 8CLCs using Yan et al. data as a reference. iBM-ALL represents all the iBM cells without downsampling.

EGA gene and pluripotency marker gene expression in 8CLCs

We next considered the expression of the genes used for marking the 8CLCs: *LEUTX* (Yu et al., 2022), *ZSCAN4* (Moya-Jódar et al., 2023), *TPRX1* (Mazid et al., 2022; Taubenschmid-Stowers et al., 2022), and *SLC34A2* (Yoshihara et al., 2022). All these markers, especially *LEUTX* and *ZSCAN4*, were most highly expressed in cluster 4 (Figure 3). *TPRX1* was minimally expressed in some cells in clusters 6 and 7 (mainly composed of morulae and some of the 8CLCs, respectively), and *SLC34A2* was also minimally expressed in some cells in clusters 2 and 3 (mainly composed of E5), in addition to clusters 6 and 7. Furthermore, many EGA genes (Töhönen et al., 2015), whose expression levels were also investigated in the original 8CLC studies, were highly expressed in the iBM cells. In contrast, their expression levels were lower or more heterogeneous in the other 8CLCs (Figure 4A). Some of these genes, such as *PRAMEF1*, *RFPL2*, and *TRIM43*, were expressed at even higher levels in the iBM cells than in embryos.

Pluripotency marker genes had unique expression patterns (Figure 4B). *NANOG* and *SOX2*, whose orthologous genes are reportedly downregulated in mouse 2CLCs (Macfarlan et al., 2012), were minimally expressed in iBM cells and 8-cell-stage embryos but highly expressed in most of the other 8CLCs. Interestingly, *POU5F1* (*OCT4*), whose expression in mouse 2CLCs was unaltered at the transcriptional level (Macfarlan et al., 2012; Rodriguez-Terrones et al., 2018), was downregulated in the sorted 8CLCs of Mazid, a phenomenon not observed in the other 8CLCs or iBM cells. *SOX2* and *POU5F1* were highly expressed in morulae or later stages but minimally expressed in 8-cell-stage embryos. The expression levels of *MYC* and cell-cycle marker genes in the M-J 8CLCs were lower than those of the other 8CLCs and pre-implantation embryos, indicating that M-J 8CLCs were less proliferative (Figure S4). Notably, *DNMT3L*, *GDF3*, *MT1E*, *NLRP7*, and *UPP1* showed low expression in iBM cells and 8-cell-stage embryos but high expression in the other 8CLCs. These genes are more highly expressed in naive hPSCs than in primed hPSCs (Stirparo et al., 2018), and this could also be related to the original cell type of the 8CLCs.

DISCUSSION

In this study, we conducted an integrative analysis of 8CLCs and human pre-implantation embryos to examine their differences and similarities. We used the scRNA-seq data from five different 8CLC studies and integrated them with two different datasets of human pre-implantation embryos. Our results revealed that the iBM cells of Yoshihara showed the highest similarity to the 8-cell-stage embryos in both reference datasets (Yan and Petropoulos), while the other 8CLC populations were more heterogeneous, with lower expression of EGA genes and higher expression of naive pluripotency markers. The cell type annotation of the 8CLCs using the scRNA-seq data of human pre-implantation embryos as references supported these findings.

Taubenschmid-Stowers et al. and Moya-Jódar et al. reported that 8CLCs emerged in naive PSC culture conditions (PXGL and 5iLAF, respectively). Mazid et al. explored the existence of 8CLCs in various naive PSC culture conditions (Guo et al., 2021; Liu et al., 2020; Yu et al., 2021) and found that small 8CLC populations were detected in cultures grown in PXGL (1.7% of the cells), 5iLA (0.7%), t2iLGö (0.5%), and RSeT (0.2%), with various expression levels of EGA genes. Our results showed that T-S 8CLCs showed higher similarity to 8-cell-stage embryos than M-J 8CLCs, indicating that the emergence of 8CLCs highly depends on the naive PSC culture conditions.

Although sorted and unsorted 8CLCs of Mazid showed similar expression patterns, the sorted 8CLCs showed

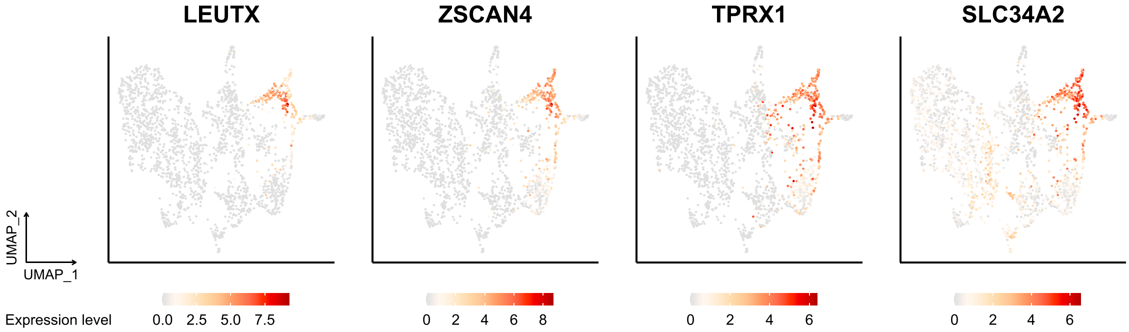

Figure 3. UMAP plots represent the expression levels of *LEUTX*, *ZSCAN4*, *TPRX1*, and *SLC34A2*

greater expression of several EGA genes, such as *KDM4E* and *KLF17*, and lower expression of *POU5F1*, *NANOG*, and *SOX2*. This suggests that sorting by *TPRX1* expression successfully enriched the cells resembling 8-cell-stage embryos. Yu et al. increased the 8CLC population by adding small molecular compounds, but these ci8CLCs were not assessed by scRNA-seq. Chemical induction may have increased not only the number of 8CLCs but also their EGA gene expression.

The iBM cells showed the highest similarity with 8-cell-stage embryos at the transcriptional level, but the developmental potency of iBM cells remains to be studied. Furthermore, before iBM cells can be used as a model to study EGA genes, the culture conditions for maintaining these cells' characteristics must be optimized. The culture conditions developed in the 8CLC studies other than that of Yoshihara et al. might be useful in this regard. However, even without established culture conditions to maintain the 8CLC phenotype *in vitro*, it is straightforward to assess the roles of individual EGA genes, especially transcription factors, by performing CRISPR-Cas9 knockouts in hPSCs before DUX4 induction and by assessing the properties of such cells after induction.

This study has several limitations. First, we integrated the scRNA-seq datasets from different studies performed using different methodologies and platforms. Although we employed rigorous data integration techniques to mitigate this issue and showed that our results were robust across various parameters, some biases might still persist. Second, our study focused primarily on gene expression, but epigenetic features such as chromatin accessibility might also play an important role in determining 8CLC characteristics. In particular, DUX4 acts as a potent enhancer activator and chromatin modifier (Vuoristo et al., 2022). Single-cell multi-omics approaches combining scRNA-seq with scATAC-seq may better clarify the differences and similarities of 8CLCs.

Despite these limitations, our study provides a timely comparison of the transcriptional landscape of 8CLCs and their similarity to pre-implantation embryos. We suggest that these initial observations on the transcriptomic differences between different 8CLC models will stimulate further in-depth research into the properties of the identified cells, their induction mechanisms and differentiation pathways, and the roles of the genes expressed in early blastomeres and implicated in EGA.

EXPERIMENTAL PROCEDURES

Resource availability

Corresponding author

Further information and requests for resources should be directed to and will be fulfilled by the corresponding authors, Masahito Yoshihara (masahito.yoshihara@chiba-u.jp) and Juha Kere (juha.kere@ki.se).

Materials availability

This study did not generate new unique reagents.

Data and code availability

This paper does not report original code. Any additional information required to reanalyze the data reported in this paper is available from the corresponding authors upon request. Data can be explored and visualized using an interactive web tool: https://my0916.shinyapps.io/8CLC-Atlas, generated by the R package ShinyCell (v2.1.0) (Ouyang et al., 2021).

Data acquisition

We used the following processed read count datasets of the scRNA-seq data of 8CLCs: NCBI Gene Expression Omnibus database under the accession numbers GSM5389327 (Taubenschmid-Stowers et al., 2022), GSM5939549 (Yu et al., 2022), GSM5931737 (Moya-Jódar et al., 2023), ArrayExpress database under the accession number E-MTAB-10581 (Yoshihara et al., 2022), and https://figshare.com/s/34110eebb58462a79dd5, https://figshare.com/s/a1b03a1463865b8a56c8 (Mazid et al., 2022). The scRNA-seq raw read datasets of human pre-implantation embryos were retrieved from E-MTAB-3929 (Petropoulos et al., 2016) and GSE36552 (Yan et al., 2013). These raw read datasets were remapped to the human GRCh38 reference genome using STAR (v2.5.1b) (Dobin et al., 2013; Zhao et al., 2021), and read counts were calculated using

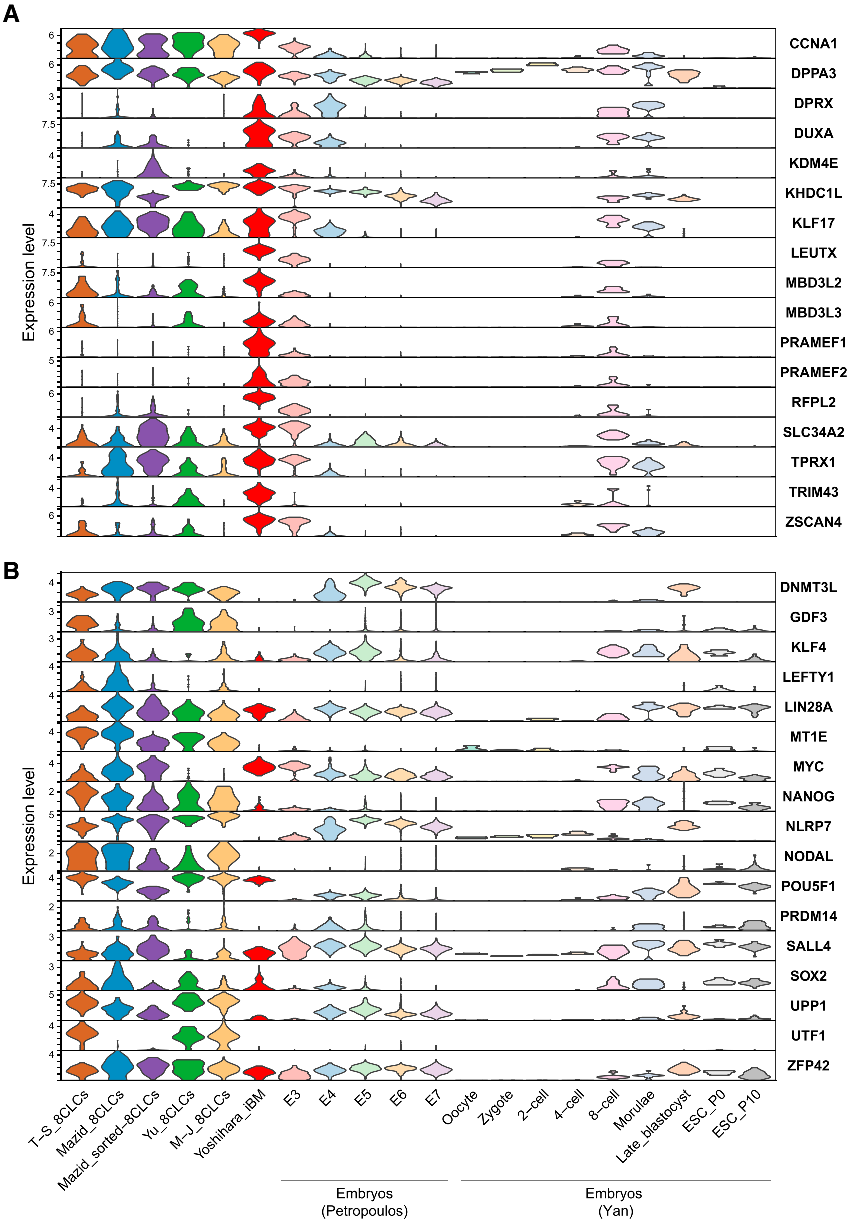

Figure 4. Violin plots show the expression levels of EGA genes, pluripotency marker genes, and naive PSC marker genes
(A) Violin plots of EGA marker genes.
(B) Violin plots of pluripotency and naive PSC marker genes. See also Figure S4.

rsem-calculate-expression of RSEM (v1.3.1) with the "–single-cell-prior" option (Li and Dewey, 2011).

Data processing to identify 8CLCs

The T-S, Yu, Yoshihara, and M-J datasets were prepared with Chromium Single-Cell 3′ library kits (10X Genomics), whereas the Mazid datasets were prepared with the droplet-based DNBelab C Series Single-Cell Library Prep set (MGI, for the stepwise e4CL-D5 data) and SMART-seq2 (for the sorted 8CLCs). The T-S and Yu datasets were processed according to the available scripts (https://github.com/rargelaguet/DUX4_8CLC_hESCs and https://github.com/ChenManqi2/ci8CLC_scripts), and the clustering process was replicated to identify the 8CLC populations. The M-J dataset was processed according to their methods, and the 8CLCs comprised the same proportion of cells (1.7%). In the Mazid datasets, 8CLCs were specified as cell names.

Data integration and comparison

The numbers of 8CLCs differed greatly between datasets: 1,528 in the Yoshihara dataset and only 28–78 in the other datasets. For comparison, we downsampled the Yoshihara dataset by randomly picking 70 cells. Genes that were commonly identified in all datasets were assessed. Log-normalized counts were calculated using the computeSumFactors function in the R package scran (v1.24.1), followed by per-batch scaling normalization using the multiBatchNorm function in the R package batchelor (v1.12.3)

(Haghverdi et al., 2018). All datasets were integrated with the fastMNN approach via SeuratWrappers (v0.3.1), using the 2,000 most highly variable genes. Cell clustering was performed with the FindNeighbors (using the top 20, 30, or 40 MNN-corrected PCs) and FindClusters (resolution = 0.2, 0.4, or 0.6) functions in the R package Seurat (v4.3.0). The top 30 PCs were selected for subsequent analysis because no remarkable difference was found by including more PCs. For the random downsampling analysis, 70 iBM cells were picked 100 times with a random seed of 123. Cell clustering was performed with resolution = 0.2–0.6, and the highest resolution values resulting in 10 or fewer clusters were selected. Then the proportion of cells clustered with the E3 or 8-cell-stage cells was calculated. Cell type annotation was conducted with the R package SingleR (v.2.0.0) (Aran et al., 2019) using the human embryo datasets (Petropoulos et al., 2016; Yan et al., 2013) as references. The scRNA-seq dataset of naive and primed hESCs was obtained from E-MTAB-6819 (Messmer et al., 2019). Cell-cycle scores (S score and G2M score) were calculated using the CellCycleScoring function of the Seurat.

SUPPLEMENTAL INFORMATION

Supplemental information can be found online at https://doi.org/10.1016/j.stemcr.2023.06.009.

ACKNOWLEDGMENTS

The authors thank Dr. Cheng Zhao for his assistance in the analysis. This work was supported by the Japan Society for the Promotion of Science Grants-in-Aid for Scientific Research (KAKENHI) grant number 22K20622 to M.Y. and by the Jane and Aatos Erkko Foundation, Sigrid Jusélius Foundation, Swedish Research Council, and Association Liv och Hälsa (Finland) to J.K.

AUTHOR CONTRIBUTIONS

M.Y. and J.K. conceived the study. M.Y. designed and performed the analyses, and M.Y. wrote the manuscript with J.K.

CONFLICT OF INTERESTS

The authors declare no competing interests.

Received: January 23, 2023
Revised: June 20, 2023
Accepted: June 21, 2023
Published: July 20, 2023

REFERENCES

Aran, D., Looney, A.P., Liu, L., Wu, E., Fong, V., Hsu, A., Chak, S., Naikawadi, R.P., Wolters, P.J., Abate, A.R., et al. (2019). Reference-based analysis of lung single-cell sequencing reveals a transitional profibrotic macrophage. Nat. Immunol. *20*, 163–172.

Bosnakovski, D., Xu, Z., Gang, E.J., Galindo, C.L., Liu, M., Simsek, T., Garner, H.R., Agha-Mohammadi, S., Tassin, A., Coppée, F., et al. (2008). An isogenetic myoblast expression screen identifies DUX4-mediated FSHD-associated molecular pathologies. EMBO J. *27*, 2766–2779.

Dobin, A., Davis, C.A., Schlesinger, F., Drenkow, J., Zaleski, C., Jha, S., Batut, P., Chaisson, M., and Gingeras, T.R. (2013). STAR: ultrafast universal RNA-seq aligner. Bioinformatics *29*, 15–21.

Genet, M., and Torres-Padilla, M.E. (2020). The molecular and cellular features of 2-cell-like cells: a reference guide. Development *147*.

Guo, G., Stirparo, G.G., Strawbridge, S.E., Spindlow, D., Yang, J., Clarke, J., Dattani, A., Yanagida, A., Li, M.A., Myers, S., et al. (2021). Human naive epiblast cells possess unrestricted lineage potential. Cell Stem Cell *28*, 1040–1056.e6.

Guo, G., von Meyenn, F., Rostovskaya, M., Clarke, J., Dietmann, S., Baker, D., Sahakyan, A., Myers, S., Bertone, P., Reik, W., et al. (2017). Epigenetic resetting of human pluripotency. Development *144*, 2748–2763.

Haghverdi, L., Lun, A.T.L., Morgan, M.D., and Marioni, J.C. (2018). Batch effects in single-cell RNA-sequencing data are corrected by matching mutual nearest neighbors. Nat. Biotechnol. *36*, 421–427.

Hendrickson, P.G., Doráis, J.A., Grow, E.J., Whiddon, J.L., Lim, J.W., Wike, C.L., Weaver, B.D., Pflueger, C., Emery, B.R., Wilcox, A.L., et al. (2017). Conserved roles of mouse DUX and human DUX4 in activating cleavage-stage genes and MERVL/HERVL retrotransposons. Nat. Genet. *49*, 925–934.

Kagawa, H., Javali, A., Khoei, H.H., Sommer, T.M., Sestini, G., Novatchkova, M., Scholte Op Reimer, Y., Castel, G., Bruneau, A., Maenhoudt, N., et al. (2022). Human blastoids model blastocyst development and implantation. Nature *601*, 600–605.

Li, B., and Dewey, C.N. (2011). RSEM: accurate transcript quantification from RNA-Seq data with or without a reference genome. BMC Bioinf. *12*, 323.

Liu, X., Ouyang, J.F., Rossello, F.J., Tan, J.P., Davidson, K.C., Valdes, D.S., Schröder, J., Sun, Y.B.Y., Chen, J., Knaupp, A.S., et al. (2020). Reprogramming roadmap reveals route to human induced trophoblast stem cells. Nature *586*, 101–107.

Macfarlan, T.S., Gifford, W.D., Driscoll, S., Lettieri, K., Rowe, H.M., Bonanomi, D., Firth, A., Singer, O., Trono, D., and Pfaff, S.L. (2012). Embryonic stem cell potency fluctuates with endogenous retrovirus activity. Nature *487*, 57–63.

Mazid, M.A., Ward, C., Luo, Z., Liu, C., Li, Y., Lai, Y., Wu, L., Li, J., Jia, W., Jiang, Y., et al. (2022). Rolling back human pluripotent stem cells to an eight-cell embryo-like stage. Nature *605*, 315–324.

Messmer, T., von Meyenn, F., Savino, A., Santos, F., Mohammed, H., Lun, A.T.L., Marioni, J.C., and Reik, W. (2019). Transcriptional Heterogeneity in Naive and Primed Human Pluripotent Stem Cells at Single-Cell Resolution. Cell Rep. *26*, 815–824.e4.

Moya-Jódar, M., Ullate-Agote, A., Barlabé, P., Rodríguez-Madoz, J.R., Abizanda, G., Barreda, C., Carvajal-Vergara, X., Vilas-Zornoza, A., Romero, J.P., Garate, L., et al. (2023). Revealing cell populations catching the early stages of human embryo development in naive pluripotent stem cell cultures. Stem Cell Rep. *18*, 64–80.

Ouyang, J.F., Kamaraj, U.S., Cao, E.Y., and Rackham, O.J.L. (2021). ShinyCell: simple and sharable visualization of single-cell gene expression data. Bioinformatics *37*, 3374–3376.

Petropoulos, S., Edsgärd, D., Reinius, B., Deng, Q., Panula, S.P., Codeluppi, S., Plaza Reyes, A., Linnarsson, S., Sandberg, R., and Lanner, F. (2016). Single-Cell RNA-Seq Reveals Lineage and

X Chromosome Dynamics in Human Preimplantation Embryos. Cell *165*, 1012–1026.

Rodriguez-Terrones, D., Gaume, X., Ishiuchi, T., Weiss, A., Kopp, A., Kruse, K., Penning, A., Vaquerizas, J.M., Brino, L., and Torres-Padilla, M.E. (2018). A molecular roadmap for the emergence of early-embryonic-like cells in culture. Nat. Genet. *50*, 106–119.

Stirparo, G.G., Boroviak, T., Guo, G., Nichols, J., Smith, A., and Bertone, P. (2018). Integrated analysis of single-cell embryo data yields a unified transcriptome signature for the human pre-implantation epiblast. Development *145*.

Taubenschmid-Stowers, J., Rostovskaya, M., Santos, F., Ljung, S., Argelaguet, R., Krueger, F., Nichols, J., and Reik, W. (2022). 8C-like cells capture the human zygotic genome activation program in vitro. Cell Stem Cell *29*, 449–459.e6.

Theunissen, T.W., Powell, B.E., Wang, H., Mitalipova, M., Faddah, D.A., Reddy, J., Fan, Z.P., Maetzel, D., Ganz, K., Shi, L., et al. (2014). Systematic identification of culture conditions for induction and maintenance of naive human pluripotency. Cell Stem Cell *15*, 524–526.

Töhönen, V., Katayama, S., Vesterlund, L., Jouhilahti, E.M., Sheikhi, M., Madissoon, E., Filippini-Cattaneo, G., Jaconi, M., Johnsson, A., Bürglin, T.R., et al. (2015). Novel PRD-like homeodomain transcription factors and retrotransposon elements in early human development. Nat. Commun. *6*, 8207.

Vuoristo, S., Bhagat, S., Hydén-Granskog, C., Yoshihara, M., Gawriyski, L., Jouhilahti, E.M., Ranga, V., Tamirat, M., Huhtala, M., Kirjanov, I., et al. (2022). DUX4 is a multifunctional factor priming human embryonic genome activation. iScience *25*, 104137.

Yan, L., Yang, M., Guo, H., Yang, L., Wu, J., Li, R., Liu, P., Lian, Y., Zheng, X., Yan, J., et al. (2013). Single-cell RNA-Seq profiling of human preimplantation embryos and embryonic stem cells. Nat. Struct. Mol. Biol. *20*, 1131–1139.

Yanagida, A., Spindlow, D., Nichols, J., Dattani, A., Smith, A., and Guo, G. (2021). Naive stem cell blastocyst model captures human embryo lineage segregation. Cell Stem Cell *28*, 1016–1022.e4.

Yoshihara, M., Kirjanov, I., Nykänen, S., Sokka, J., Weltner, J., Lundin, K., Gawriyski, L., Jouhilahti, E.M., Varjosalo, M., Tervaniemi, M.H., et al. (2022). Transient DUX4 expression in human embryonic stem cells induces blastomere-like expression program that is marked by SLC34A2. Stem Cell Rep. *17*, 1743–1756.

Yu, L., Wei, Y., Duan, J., Schmitz, D.A., Sakurai, M., Wang, L., Wang, K., Zhao, S., Hon, G.C., and Wu, J. (2021). Blastocyst-like structures generated from human pluripotent stem cells. Nature *591*, 620–626.

Yu, X., Liang, S., Chen, M., Yu, H., Li, R., Qu, Y., Kong, X., Guo, R., Zheng, R., Izsvák, Z., et al. (2022). Recapitulating early human development with 8C-like cells. Cell Rep. *39*, 110994.

Zhao, C., Reyes, A.P., Schell, J.P., Weltner, J., Ortega, N.M., Zheng, Y., Björklund, Å.K., Rossant, J., Fu, J., Petropoulos, S., et al. (2021). Reprogrammed blastoids contain amnion-like cells but not trophectoderm. Preprint at bioRxiv. https://doi.org/10.1101/2021.05.07.442980.

Stem Cell Reports, Volume *18*

Supplemental Information

Transcriptomic differences between human 8-cell-like cells reprogrammed with different methods

Masahito Yoshihara and Juha Kere

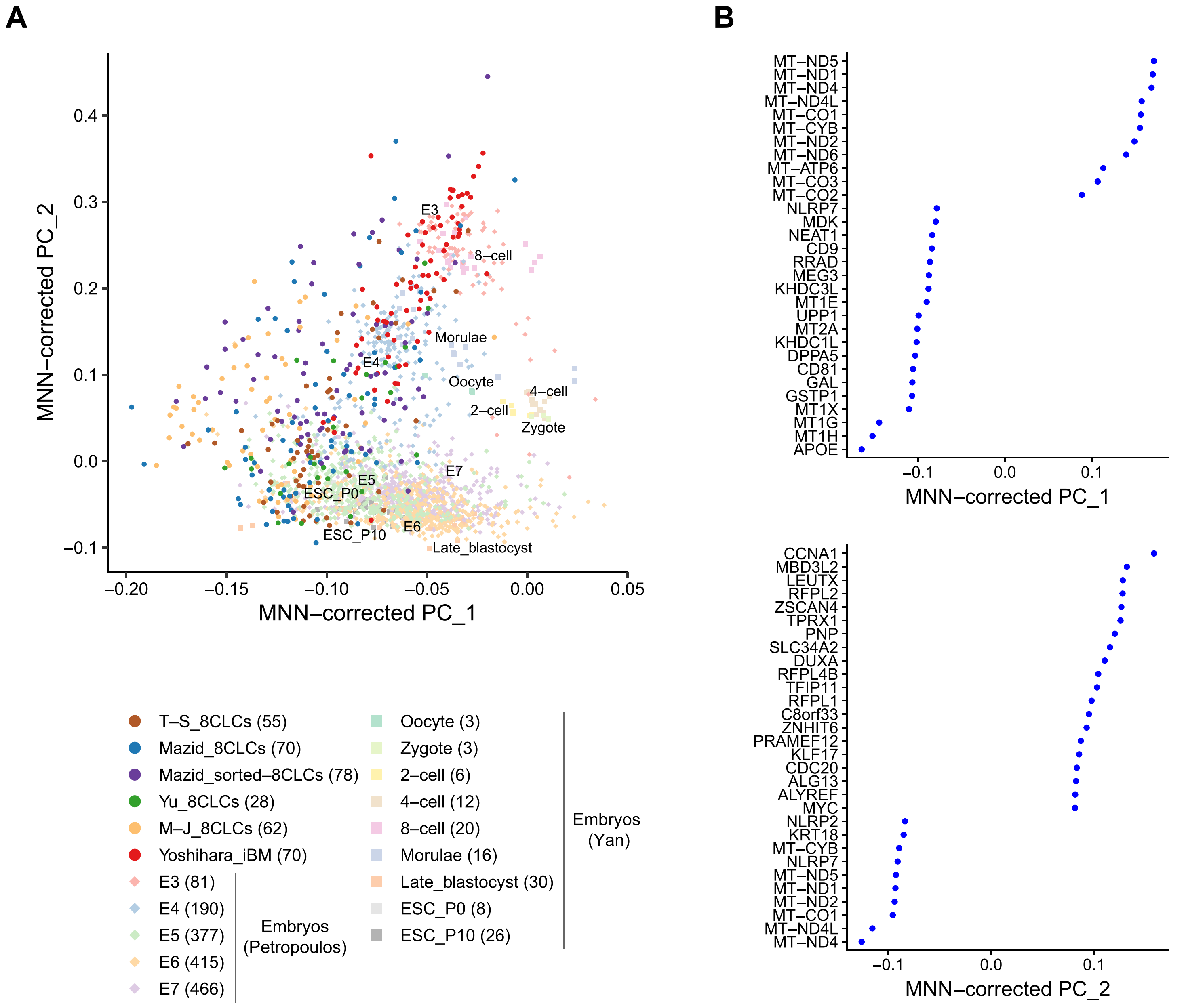

Figure S1. MNN-corrected PCA of human 8CLCs with pre-implantation embryos, related to Figure 1

(A) A PCA plot colored by the original cell types. T-S and M-J stand for Taubenschmid-Stowers and Moya-Jódar, respectively. E3–7, embryonic day 3–7; P0, passage 0; P10, passage 10. Numbers in parentheses represent the numbers of analyzed cells.

(B) Top 30 genes contributing to the MNN-corrected principal components (PCs) 1 and 2.

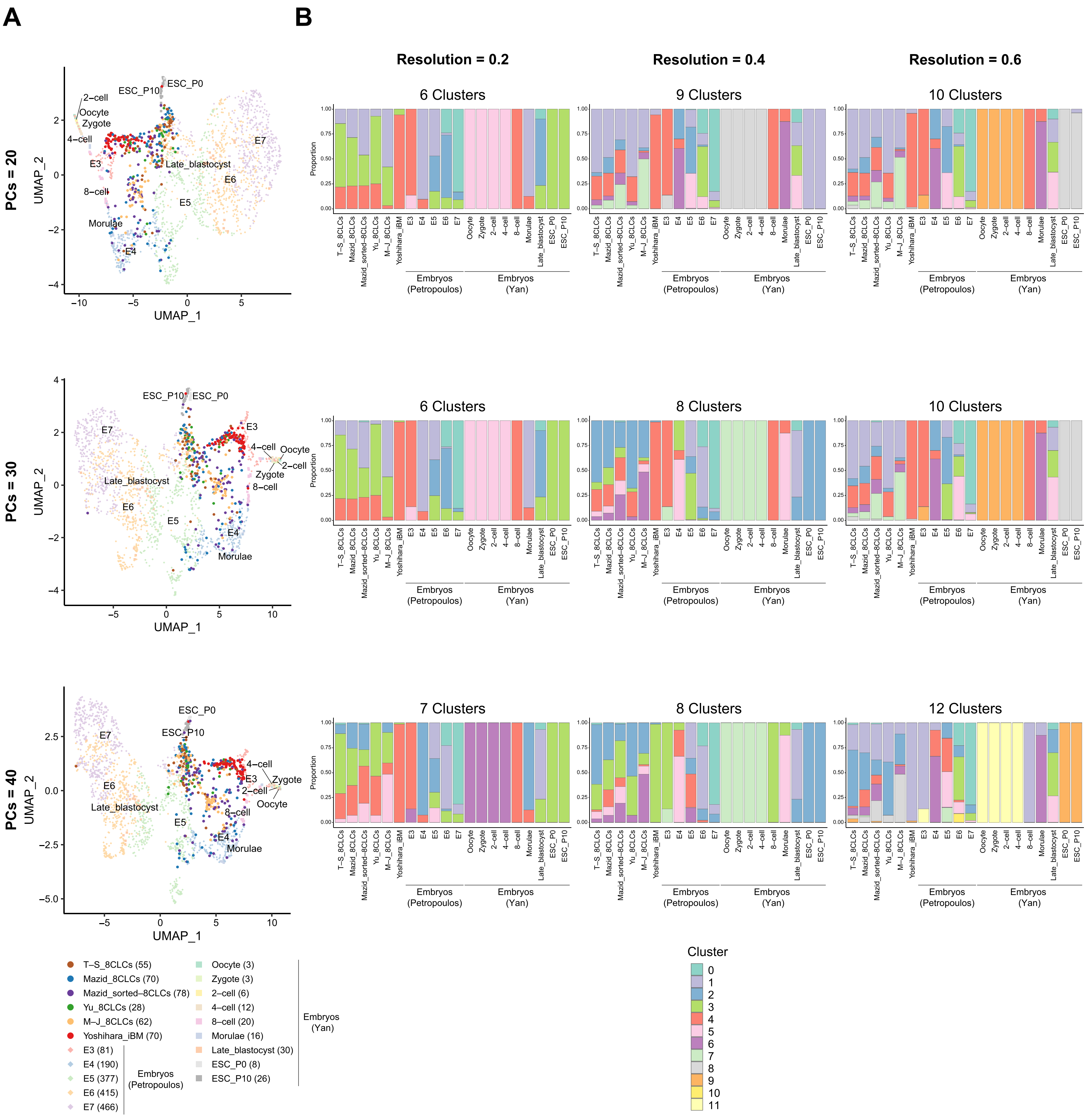

Figure S2. Unsupervised clustering of human 8CLCs and pre-implantation embryos with different parameters, related to Figure 1

(A) UMAP plots colored by the original cell types. UMAP was generated using the top 20, 30, or 40 MNN-corrected PCs.

(B) Bar plots show the proportion of each cluster in each cell type. Clustering was performed with different resolutions (0.2, 0.4, or 0.6) for each parameter shown in A.

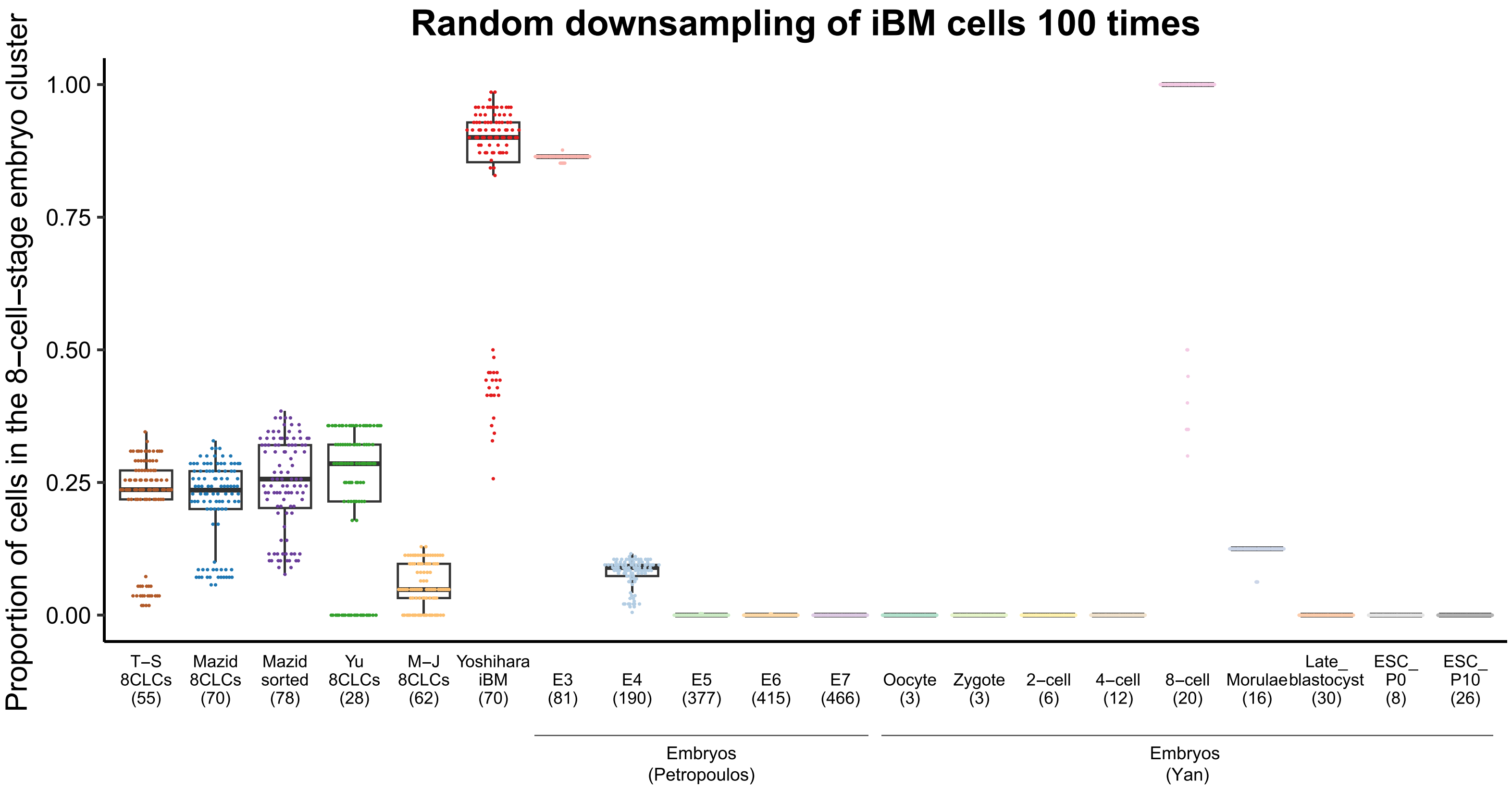

Figure S3. The proportion of cells clustered with 8-cell-stage embryos after random downsampling of iBM cells 100 times, related to Figure 1

iBM cells of Yoshihara were randomly picked 100 times and the proportion of cells clustered with the E3 or 8-cell-stage cells was calculated. Each dot represents one iteration.

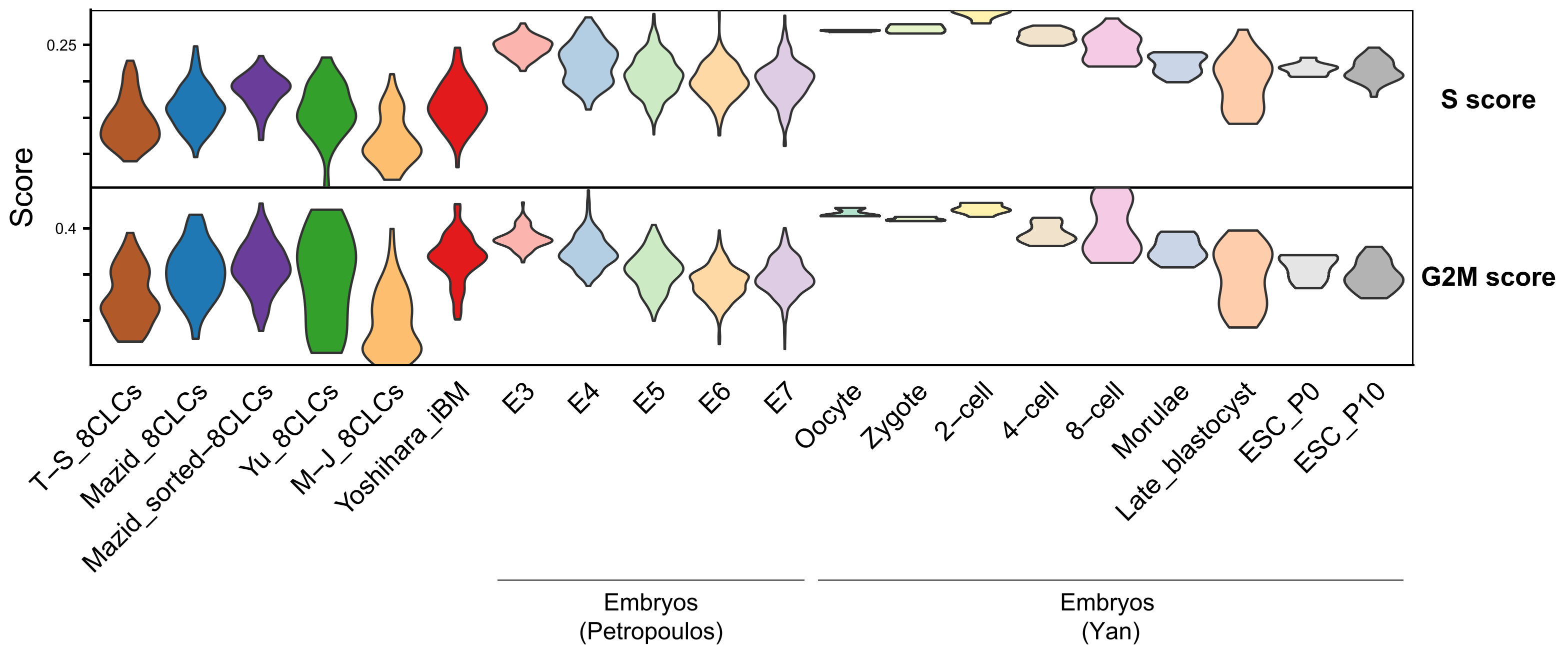

Figure S4. Violin plots show the cell cycle scores, related to Figure 4
